# Supplementary figures and images for: Cortical mechanisms of across-ear speech integration investigated using functional near-infrared spectroscopy (fNIRS)
Source: PLoS One. 2024 Sep 18;19(9):e0307158. doi: 10.1371/journal.pone.0307158 (PMC11410267; doi:10.1371/journal.pone.0307158)

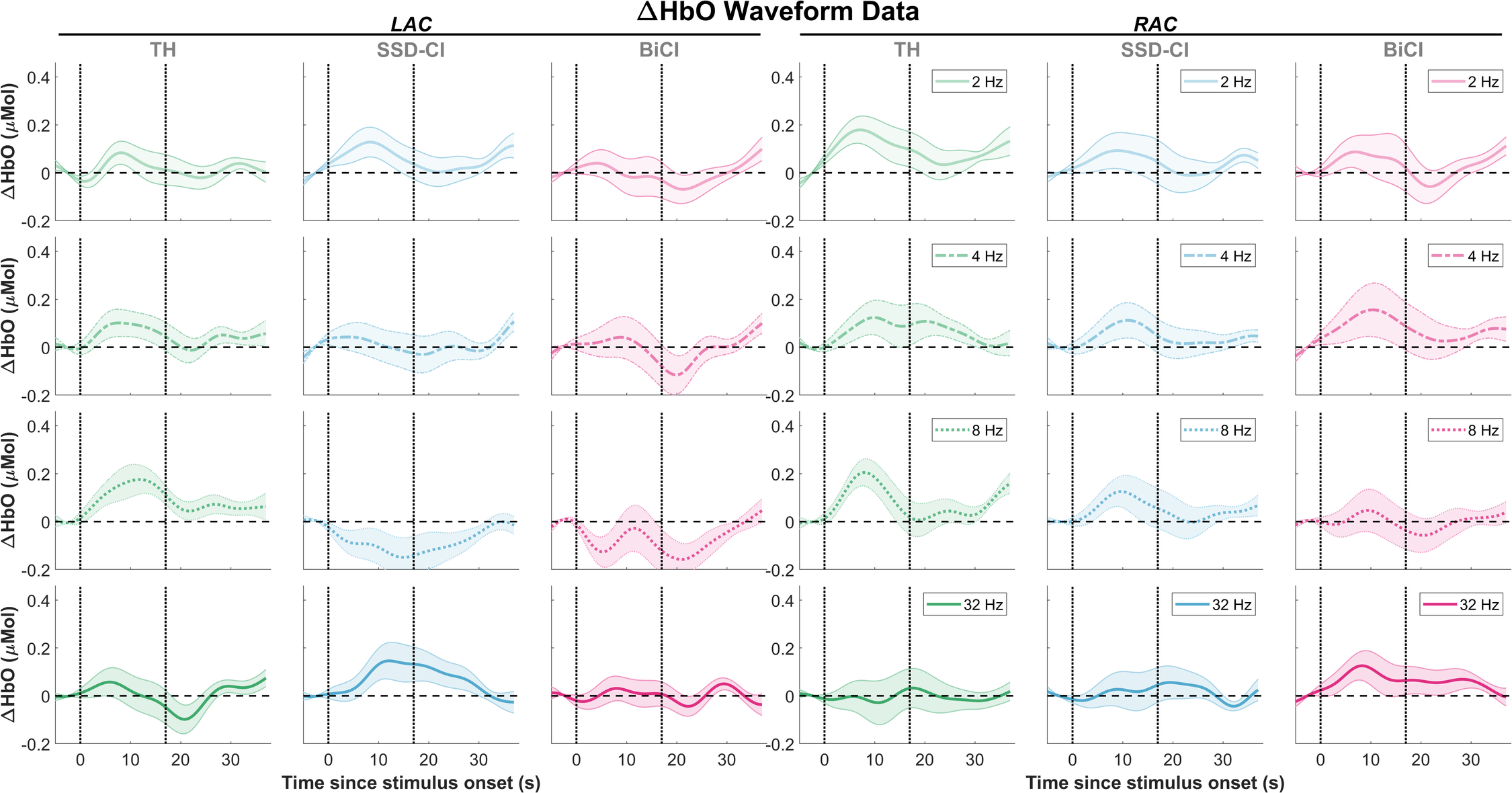

Supplement: S1 Fig — Each plot contains waveforms at one alternating rate for one speech condition. Linear traces indicate mean response amplitude, and shaded regions correspond to the standard error of the mean response amplitude. Columns correspond to the three speech conditions, and data from a single cortical ROI is contained under the solid horizontal line with corresponding ROI label. Within each plot, vertical dotted lines correspond to stimulus onset and offset. (TIF) [file pone.0307158.s001.tif]

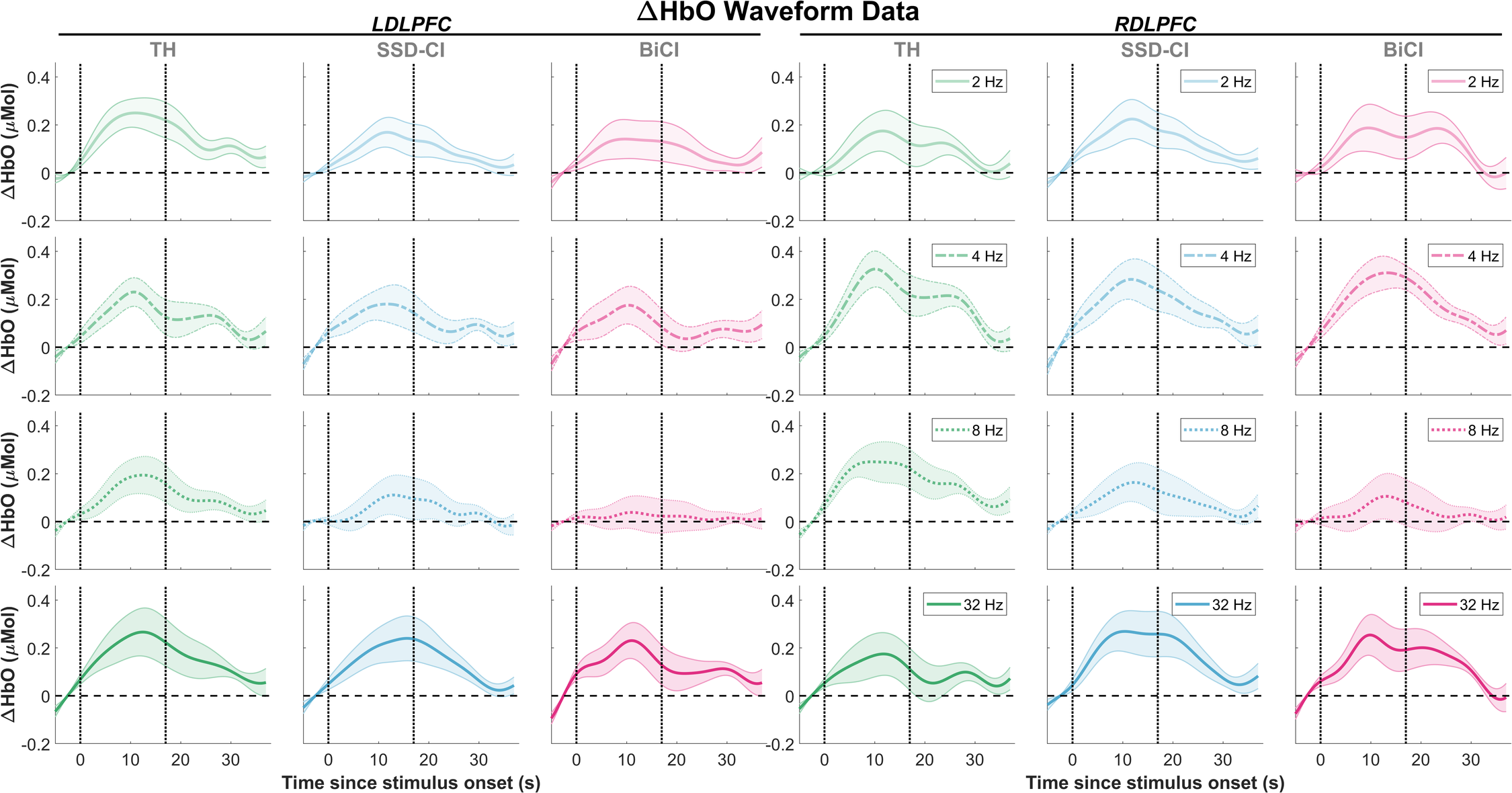

Supplement: S2 Fig — Each plot contains waveforms at one alternating rate for one speech condition. Linear traces indicate mean response amplitude, and shaded regions correspond to the standard error of the mean response amplitude. Columns correspond to the three speech conditions, and data from a single cortical ROI is contained under the solid horizontal line with corresponding ROI label. Within each plot, vertical dotted lines correspond to stimulus onset and offset. (TIF) [file pone.0307158.s002.tif]

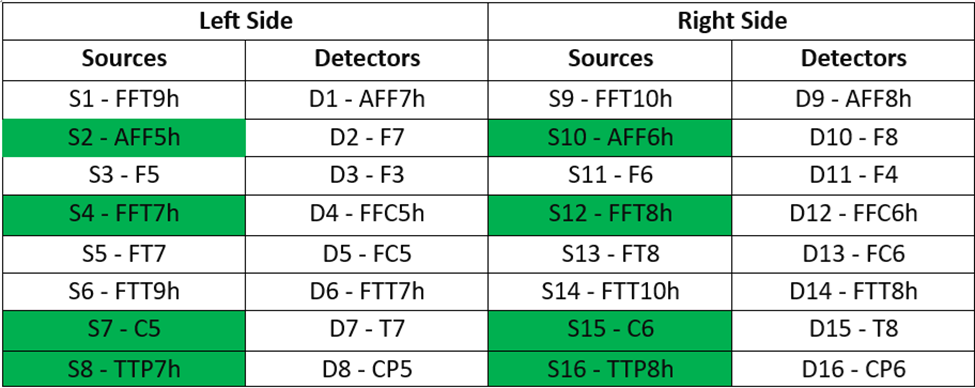

Supplement: S1 Table — (TIF) [file pone.0307158.s003.tif]

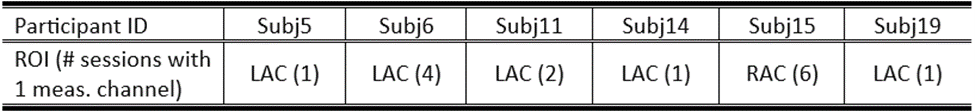

Supplement: S2 Table — (TIF) [file pone.0307158.s004.tif]

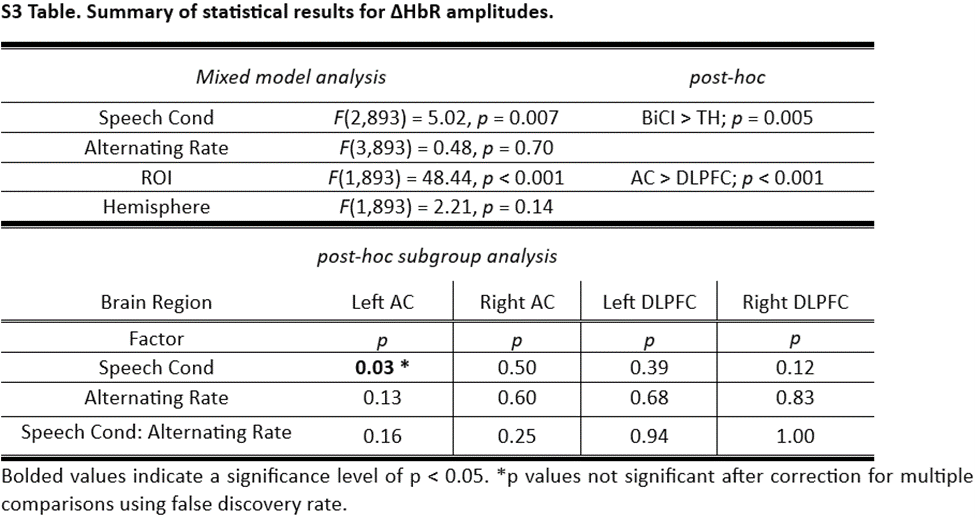

Supplement: S3 Table — (TIF) [file pone.0307158.s005.tif]
