# Supplementary material for: Cortical mechanisms of across-ear speech integration investigated using functional near-infrared spectroscopy (fNIRS)
Source: PLoS One. 2024 Sep 18;19(9):e0307158. doi: 10.1371/journal.pone.0307158 (PMC11410267; doi:10.1371/journal.pone.0307158)
Supplement: S1 File — This is the document containing additional information pertinent to the study for readers to reference. Open Science Framework file-sharing link: https://osf.io/xdmwy/?view_only=2d17c98b9ae34ee9864b359c07bf0332. (DOCX) [file pone.0307158.s006.docx]

Supplementary materials

# **fNIRS data collection**

The optode placement on the International 10-10 system was shown in S1 Table. The NIRScap was again positioned in accordance with the 10-10 system, with Cz halfway between the naison and inion and halfway between the two pre-auricular points. Fp1 was positioned at 10% of the naison-inion distance (approximately a few cm above the eyebrows). To ensure that the cap was fixed in place for the duration of testing, participants wore a chest strap that was attached to the NIRSCap. The fNIRS system was then calibrated to assess the gains of light intensity at the APD detectors. Channels showing “poor” or “lost” light intensity likely indicated interposition of hair between the optode and scalp, causing mispositioning of the optode or interference with the photon path. To rectify this issue, optodes were removed from the NIRSCap and interposed hair was brushed aside to allow for improved scalp contact when the optode was replaced. This procedure was repeated until all channels showed at least “acceptable” light intensity.

## **Rationale for Short Channels**

The optode arrangement in this experiment included both 30 mm “measurement” channels and 8 mm “short” channels. This experiment employed the GLM-PCA method during data analysis to reduce systemic noise in the fNIRS data. A key assumption of the GLM-PCA method is that fNIRS data contains both extracerebral and intracerebral components, and by reducing extracerebral components the signal quality is improved [1–3]. Inclusion of short channels permitted for direct measurement of predominantly extracerebral vascular dynamics: Due to near-infrared photon reflection and dispersion through different tissues, the photon path between source and detector follows a U-shaped path. By shortening the distance between source and detector, the photon trajectory becomes shallower [4,5], thereby excluding intracerebral vascular dynamics. Implementation of data from the short channels to reduce systemic noise in the fNIRS data from measurement channels is described in further detail below and in the main manuscript.

**S1 Table.** **Placement of fNIRS sources (S, n=16) and detector (D, n=16) on the 10-10 system.**

| **Left Side** | | **Right Side** | |
| --- | --- | --- | --- |
| **Sources** | **Detectors** | **Sources** | **Detectors** |
| S1 - FFT9h | D1 - AFF7h | S9 - FFT10h | D9 - AFF8h |
| S2 - AFF5h | D2 - F7 | S10 - AFF6h | D10 - F8 |
| S3 - F5 | D3 - F3 | S11 - F6 | D11 - F4 |
| S4 - FFT7h | D4 - FFC5h | S12 - FFT8h | D12 - FFC6h |
| S5 - FT7 | D5 - FC5 | S13 - FT8 | D13 - FC6 |
| S6 - FTT9h | D6 - FTT7h | S14 - FTT10h | D14 - FTT8h |
| S7 - C5 | D7 - T7 | S15 - C6 | D15 - T8 |
| S8 - TTP7h | D8 - CP5 | S16 - TTP8h | D16 - CP6 |

The green-shaded sources mark the optodes where short-channel detectors were connected.

# **fNIRS data analyses**

The pre-processing pipeline was described in the manuscript. Here we provide further details about critical steps.

**Channel rejection.** Channels were rejected based on their scalp coupling index, or SCI [6], which reveals the sensitivity in measuring heartbeat signals in each channel. Instead of a cutoff threshold of 0.15 in [1], in this study, the cutoff threshold was set to 0.25 to ensure that each participant had at least 4 out of 8 short channels remaining for further analysis. The mean ± SD percentages of measurement channels excluded were 5.01% ± 7.19%; for short channels, these values were 12.36% ± 14.96%.

**Wavelet analysis.** In the wavelet decomposition algorithm from [7], wavelet coefficients further than 0.1 times the interquartile range below the first quartile or above the third quartile were excluded. This was the same value used in [8] and is commonly used in the fNIRS literature.

**GLM-PCA method for reducing systemic noise.** A generalized linear model-principal component analysis (GLM-PCA) method was applied to reduce the extracerebral components in the HbO and HbR responses, separately, in the measurement (30 mm) channels. For fNIRS signals in both short (8 mm) and measurement channels, a third-order Butterworth band-pass filter (cutoff frequencies 0.01 to 1.5 Hz) was applied to remove low- and high-frequency physiologic noise. A PCA was then performed on all short channels with SCI ≥ 0.25 for each data collection session from each participant. The first two principal components (PCs) from the short-channel responses were assumed to represent a “global” variance across channels and therefore needed to be removed. The first two PCs extracted from HbO and HbR short-channel response data explained 83.3% ± 4.6% and 63.2% ± 9.0% of the variance, respectively, in the fNIRS signals across participants for nine sessions of data collection. The two PCs were used as regressors in a general linear model (GLM), the product of which and the corresponding coefficients from GLM were then subtracted from ∆HbO and ∆HbR, separately, in each channel.

**Block-average and calculation of the amplitude for each ROI.** A third-order Butterworth bandpass filter (0.01 – 0.09 Hz) was applied after the GLM-PCA method, and ∆HbO and ∆HbR in the measurement channels were then block-averaged: baseline averages from the 5 seconds prior to the onset of each stimulus block were subtracted out in this calculation, and individual blocks with values outside ± 2.5 SDs of all blocks to be averaged were excluded. To compute block-averaged responses in each ROI, the means of block-average responses across channels that clustered into ROIs were taken, for each participant in each condition. Prior research has shown that on average, the time to peak fNIRS hemodynamic response is at least 5 seconds after stimulus onset [9]. To isolate stimulus-evoked brain activation in a channel, the peak of the block-averaged ∆HbO amplitude of in each channel between 5 and 19 s after the onset of the stimulus was identified. Then, the ∆HbO amplitude across a 10 s window centered on the identified peak was averaged to obtain the activation level to a particular stimulus [10].

## **Further Details On Subject-Level Channel Rejection**

In the bilateral DLPFC, for all participants there were 3/4 or 4/4 measurement channels available in all data collection sessions. In the AC, there were 15 data collection sessions spread across 6 participants in which 2 measurement channels were rejected, with 1 remaining channel in the ROI. These findings are summarized in S2 Table. Of note, Subj6 and Subj15 had 4/9 and 6/9 recording sessions with one remaining measurement channel, respectively, in one ROI. This was most likely due to hair-related artifacts as both participants were female. The presence of only one measurement channel could have negatively impacted data quality; however, we elected not to exclude these participants because they had robust data in the remaining 3 ROIs.

**S2 Table.** Participants with 1 remaining measurement channel in either auditory cortex following channel exclusion protocols.

| Participant ID | Subj5 | Subj6 | Subj11 | Subj14 | Subj15 | Subj19 |
| --- | --- | --- | --- | --- | --- | --- |
| ROI (# sessions with 1 meas. channel) | LAC (1) | LAC (4) | LAC (2) | LAC (1) | RAC (6) | LAC (1) |

# **Results**

## **Effect of testing order on speech intelligibility performance**

As a proxy for examining the potential effects of learning on intelligibility performance, we examined whether testing occurred on Day 1 or Day 2 and the interval between Day 1 and Day 2 on intelligibility scores. The average interval between Day 1 and Day 2 was 4 ± 5.6 days (range 1 – 19 days). An ART analysis was performed on the RAU data with testing day (Day 1, Day 2) and interval between Day 1 and Day 2 as fixed factors and participant as a random factor. Results found no difference in performance between participants who were tested on Day 1 vs. Day 2 (*F*(1,14) = 0.38, *p* = 0.55). For participants tested on Day 2, there was no effect of delay between Day 1 and Day 2 on speech intelligibility performance (*F*(1,14) = 0.61, *p* = 0.66), suggesting a negligible effect of learning on speech intelligibility scores.

### **fNIRS results**

S1 Fig and S2 Fig plot the group average and standard error of the mean (SEM, shaded errors) of ΔHbO responses in the auditory cortex (AC) and dorsolateral prefrontal cortex (DLPFC), respectively.

Statistical analyses were conducted on ΔHbO and ΔHbR measures, separately, to examine differences in group mean response amplitudes among the four ROIs. Statistical results for ΔHbO were reported in the manuscript. S3 Table summarizes the results for ΔHbR. There were significant main effects of speech condition and ROI, but no significant effects of alternating rate or hemisphere. All combinations of interactions between the fixed factors were non-significant. Post hoc analyses revealed significant differences between BiCI and TH conditions (*p* = 0.005) with more positive response amplitudes in BiCI conditions compared to the TH conditions. Unlike with ΔHbO data, the AC showed higher ΔHbR response amplitudes compared to the DLPFC, likely due to the overall less negative ΔHbR responses observed in the AC compared to the DLPFC (see **Fig 5**). Subgroup analyses found that the only significant finding was a main effect of speech condition with ΔHbR data in the left AC (*F*(2,893) = 3.60, *p_unadjusted_* = 0.03), indicating sensitivity of the left AC to intelligible speech, as addition of vocoded speech in the simulated SSD-CI and BiCI conditions reduced speech intelligibility.

**S3 Table. Summary of statistical results for ΔHbR amplitudes.**

| *Mixed model analysis* | | | *post-hoc* | |
| --- | --- | --- | --- | --- |
| Speech Cond | *F*(2,893) = 5.02, *p* = 0.007 | | BiCI > TH; *p* = 0.005 | |
| Alternating Rate | *F*(3,893) = 0.48, *p* = 0.70 | |  | |
| ROI | *F*(1,893) = 48.44, *p* < 0.001 | | AC > DLPFC; *p* < 0.001 | |
| Hemisphere | *F*(1,893) = 2.21, *p* = 0.14 | |  | |
| *post-hoc subgroup analysis* | | | | |
| Brain Region | Left AC | Right AC | Left DLPFC | Right DLPFC |
| Factor | *p* | *p* | *p* | *p* |
| Speech Cond | **0.03 *** | 0.50 | 0.39 | 0.12 |
| Alternating Rate | 0.13 | 0.60 | 0.68 | 0.83 |
| Speech Cond: Alternating Rate | 0.16 | 0.25 | 0.94 | 1.00 |

Bolded values indicate a significance level of p < 0.05. *p values not significant after correction for multiple comparisons using false discovery rate.

# **References**

1. Zhou X, Sobczak G, Mckay CM, Litovsky RY. Comparing fNIRS signal qualities between approaches with and without short channels. PLoS One [Internet]. 2020;15(12):1–18. Available from: http://dx.doi.org/10.1371/journal.pone.0244186

2. Tak S, Ye JC. Statistical analysis of fNIRS data: A comprehensive review. Neuroimage [Internet]. 2014;85:72–91. Available from: http://dx.doi.org/10.1016/j.neuroimage.2013.06.016

3. Pinti P, Scholkmann F, Hamilton A, Burgess P, Tachtsidis I. Current Status and Issues Regarding Pre-processing of fNIRS Neuroimaging Data: An Investigation of Diverse Signal Filtering Methods Within a General Linear Model Framework. Front Hum Neurosci. 2019;12(January):1–21.

4. Sato T, Nambu I, Takeda K, Aihara T, Yamashita O, Isogaya Y, et al. Reduction of global interference of scalp-hemodynamics in functional near-infrared spectroscopy using short distance probes. Neuroimage [Internet]. 2016;141:120–32. Available from: http://dx.doi.org/10.1016/j.neuroimage.2016.06.054

5. Huppert TJ, Diamond SG, Franceschini MA, Boas DA. HomER: A review of time-series analysis methods for near-infrared spectroscopy of the brain. Appl Opt. 2009;48(10).

6. Pollonini L, Olds C, Abaya H, Bortfeld H, Beauchamp MS, Oghalai JS. Auditory cortex activation to natural speech and simulated cochlear implant speech measured with functional near-infrared spectroscopy. Hear Res. 2014;309:84–93.

7. Molavi B, Dumont GA. Wavelet-based motion artifact removal for functional near-infrared spectroscopy. Physiol Meas. 2012;33(2):259–70.

8. Zhou X, Seghouane AK, Shah A, Innes-Brown H, Cross W, Litovsky R, et al. Cortical Speech Processing in Postlingually Deaf Adult Cochlear Implant Users, as Revealed by Functional Near-Infrared Spectroscopy. Trends Hear. 2018;22:1–18.

9. Handwerker DA, Ollinger JM, D’Esposito M. Variation of BOLD hemodynamic responses across subjects and brain regions and their effects on statistical analyses. Neuroimage. 2004 Apr;21(4):1639–51.

10. Zhou X, Seghouane AK, Shah A, Innes-Brown H, Cross W, Litovsky R, et al. Cortical Speech Processing in Postlingually Deaf Adult Cochlear Implant Users, as Revealed by Functional Near-Infrared Spectroscopy. Trends Hear. 2018 Jan 19;22.
