# Supplementary material for: Cortical mechanisms of across-ear speech integration investigated using functional near-infrared spectroscopy (fNIRS)
Source: PLoS One. 2024 Sep 18;19(9):e0307158. doi: 10.1371/journal.pone.0307158 (PMC11410267; doi:10.1371/journal.pone.0307158)
Supplement: Attachment — Submitted filename: Response to Reviewers_PONE-D-23-42460_final.docx [file pone.0307158.s007.docx]

**Letter in Response to Reviewer Comments Regarding PONE-D-23-42460**

*The authors greatly appreciate the reviewers’ time and insightful feedback regarding the first draft of this manuscript. We have addressed all comments as received with short narrative summaries and referenced location of changes in the manuscript. Changes made to the manuscript itself are in bolded, blue text. New citations are included in line with manuscript revisions, and are highlighted in blue, bold text in the References list.*

*In addition to revisions as outlined in the table below, the authors have also updated the format of the manuscript to adhere to PLoS ONE journal requirements. Stylistic changes made in accordance with PLoS ONE guidelines are not highlighted.*

|  | **Summary:** The manuscript investigates and assesses potential cortical signatures of across-ear integration of alternating speech. The study utilizes fNIRS technique to record brain signals and after processing found similar results to previous work with alternating speech. The manuscript is well-structured and has performed fNIRS data acquisition and analysis. However, it briefly mentions limitations, such as potential effects of exposure to sentence structures and the need for further research on the observed graphical discrepancy between behavioral and neuroimaging data. | |
| --- | --- | --- |
|  | **Reviewer 1's Comments** | **Response** |
| 1 | Which type of noise was removed by applying bandpass of 0.01–0.5 Hz? Why choose only this band? | The authors appreciate these clarifying questions. This filter was reported in error; the bandpass filter employed prior to GLM-PCA analysis was 0.01 – 1.5 Hz, not 0.01 – 0.5 Hz as written. We followed the same signal preprocessing pipeline as described in our prior study (1). This has been corrected in the updated manuscript, on page 11.  The fNIRS signal is composed of multiple components due to the reflection geometry of NIR imaging: {1} evoked neurovascular coupling, or the target signal; {2} non-evoked neurovascular coupling; {3} evoked and non-evoked systemic physiologic processes. {2} and {3} may be intra-cerebral or extra-cerebral in nature (1–3). The fNIRS data processing algorithm in this study attempts to minimize noise components of multiple origins, and several bandpass filters of different frequency ranges are applied at various stages, outlined in the Supplementary materials. The bandpass filter in question is applied to the dataset after channel rejection and wavelet decomposition, but prior to GLM-PCA. Post GLM-PCA, we used another bandpass filter (0.01 – 0.09 Hz) to further remove physiologic noise such as respirations and heartbeats, as recommended by Pinti and colleagues (3). We included detailed information in S1 File. |
| 2 | The results were discussed very briefly and description of results may help the reader to better understand. | The authors appreciate this feedback and have included a summary at the beginning of the Results, page 13, section to aid in better understanding our analyses. |
| 3 | In statistical results for speech intelligibility, multiple p–values are non-significant, that should be discussed in the results as well and need explanation. | The authors appreciate this point of clarification. We included specific references to the non-significant *p* values observed in the speech intelligibility data in the Results section, page 14. Potential explanations for the non-significant *p* values are discussed in the “Patterns in Speech Intelligibility Data and Sentence Identification” sub-section of the Discussion section. Please refer to comments on the ceiling-level scores in the TH and SSD-CI speech conditions on pages 18-19, within the Discussion section. Between-speech condition scores for TH and SSD-CI were significantly higher compared with BiCI scores across alternating rates, confirming our hypothesis about behavioral data. We thus clarified references to “ceiling-level” performance as an extension of this finding, in combination with observations that (1) within-speech condition scores were not significantly different across alternating rates for the TH and SSD-CI conditions; (2) between-speech condition scores for TH and SSD-CI conditions were not significantly different across alternating rates. |
| 4 | The study claims fNIRS data acquisition and analysis as novel contribution, the comparison of fMRI and EEG for the same experiment should be included to strengthen the claim and also to investigate what significance contribution fNIRS brought to the study. | The authors appreciate this feedback. A primary objective of this study was to establish that fNIRS can detect changes in cortical neural activity in response to vocoded and non-vocoded alternating speech. We intend to replicate the study in listeners with cochlear implants (CIs); prior research (4–7) has demonstrated that fNIRS is an ideal functional neuroimaging modality for listeners with CIs. By employing optical probes instead of electrodes (EEG) or magnetic fields (fMRI), fNIRS is less impeded by ferromagnetic and electrical components of the CI. While it is true that this is the first functional neuroimaging study to utilize the alternating speech stimulus, we do not claim that fNIRS data acquisition and analysis in and of themselves are novel and have updated the “Cortical Regions of Interest” subheading of the Introduction to appropriately convey this (page 5). Prior literature has already validated fNIRS as an imaging modality: The fNIRS response functions for both Hb molecules correlate well with the blood oxygenation level-dependent (BOLD) response in fMRI (8–10). We concur that replication of findings in alternative imaging modalities would strengthen our conclusions, but such comparisons are beyond the scope of this pilot study. We have already performed a literature review on cortical integration of dichotic stimuli which can be found in the Discussion section, under subheadings regarding AC and DLPFC response patterns (pages 19 – 22). |
| 5 | Ethical statement and IRB approval reference should be added in the manuscript. | The authors appreciate this feedback. The required revisions to the manuscript have been made to the Methods section, included under the Ethics Statement sub-heading. |

|  | **Summary:** n/a | |
| --- | --- | --- |
|  | **Reviewer 2’s comments** | **Responses** |
| 1 | The manuscript acknowledges several limitations, including the variability in fNIRS data and the absence of specific control conditions. However, these limitations are not thoroughly discussed in terms of their potential impact on the interpretation of the results. | The authors appreciate this feedback. We have included specific discussion in the Limitations section about how lack of monaural control conditions might impact interpretation of our behavioral speech intelligibility and fNIRS data as a manifestation of an across-ear, or binaural, phenomenon (page 25). In our prior version, we have discussed the impact of other identified limitations including the variability in fNIRS data on interpretation of results (pages 25 – 26) hence we did not include additional revisions. |
| 2 | Additionally, in Figure 1, it is noted that a few channels have a length of less than 30mm, indicating short channels. However, the manuscript fails to provide an explanation for why these short channels are used or why the focus is solely on channels with 30mm-separated optodes. Clarifying the rationale behind the inclusion of short channels or providing justification for focusing on specific channel configurations would enhance the understanding of the experimental setup. | The authors appreciate these points of clarification. The utility of the 8mm, or short, channels is briefly detailed in the Methods section, under the fNIRS Data Collection sub-heading. We have added additional information regarding implementation of short channels for fNIRS data analysis in the Supplemental materials and referenced this in the main manuscript (page 9). In brief, short channels are intended to record exclusively extra-cerebral signals, whereas the 30 mm measurement channels record both extra-cerebral and intra-cerebral signals. The GLM-PCA model utilizes data from the short channel as regressors to reduce extra-cerebral components in the measurement channel data. These short channels were strategically placed adjacent to cortical regions of interest to enhance noise reduction in the channels of interest (see Fig 1).  Our *a priori* regions of interest for this experiment were the bilateral DLPFC and AC. Specific channel configurations were created by selecting combinations of channels from the main montage that yielded the greatest probabilistic sensitivity in regions of the “Colin27” MNI template corresponding to the DLPFC and AC. For the DLPFC, this was Brodmann areas 45, 46 and 9; for the AC, this was Brodmann areas 41 and 42 (11). These details have been added to the relevant sub-heading in the Methods section of the main manuscript (page 9). Remaining channels in the montage were included per the operating specifications of the continuous-wave fNIRS system in our lab, and data from all channels were reviewed graphically as part of initial analyses, which were not relevant to discussions in this manuscript.  Finally, the authors have changed the figure placement for Figures 1 and 2 to improve the overall flow of the Methods section. |
| 3 | Furthermore, Figures 3, 4, and 5 could be improved in terms of visualization. The lines of the plots are too light in color, making them difficult to distinguish, and the figures appear blurred. Enhancing the contrast of the lines and ensuring better resolution would significantly improve the clarity and interpretability of the figures, thereby enhancing the overall presentation of the results. | The authors appreciate this feedback. Figure 3 was updated to be a .tif file for improved clarity on publication. The color scheme for Figure 4 was changed to improve contrast of each individual line. The weight of the line plots in Figure 5 was increased to improve contrast and visibility. |

# **References**

1.	Zhou X, Sobczak G, Mckay CM, Litovsky RY. Comparing fNIRS signal qualities between approaches with and without short channels. PLoS One [Internet]. 2020;15(12):1–18. Available from: http://dx.doi.org/10.1371/journal.pone.0244186

2.	Tak S, Ye JC. Statistical analysis of fNIRS data: A comprehensive review. Neuroimage [Internet]. 2014;85:72–91. Available from: http://dx.doi.org/10.1016/j.neuroimage.2013.06.016

3.	Pinti P, Scholkmann F, Hamilton A, Burgess P, Tachtsidis I. Current Status and Issues Regarding Pre-processing of fNIRS Neuroimaging Data: An Investigation of Diverse Signal Filtering Methods Within a General Linear Model Framework. Front Hum Neurosci. 2019;12(January):1–21.

4.	Pollonini L, Olds C, Abaya H, Bortfeld H, Beauchamp MS, Oghalai JS. Auditory cortex activation to natural speech and simulated cochlear implant speech measured with functional near-infrared spectroscopy. Hear Res. 2014;309:84–93.

5.	Olds C, Pollonini L, Abaya H, Larky J, Loy M, Bortfeld H, et al. Cortical activation patterns correlate with speech understanding after cochlear implantation. Ear Hear. 2016;37(3):e160–72.

6.	Zhou X, Seghouane AK, Shah A, Innes-Brown H, Cross W, Litovsky R, et al. Cortical Speech Processing in Postlingually Deaf Adult Cochlear Implant Users, as Revealed by Functional Near-Infrared Spectroscopy. Trends Hear. 2018;22:1–18.

7.	Harrison SC, Lawrence R, Hoare DJ, Wiggins IM, Hartley DEH. Use of Functional Near-Infrared Spectroscopy to Predict and Measure Cochlear Implant Outcomes: A Scoping Review. Brain Sci. 2021 Oct 28;11(11):1439.

8.	Scholkmann F, Kleiser S, Metz AJ, Zimmermann R, Mata Pavia J, Wolf U, et al. A review on continuous wave functional near-infrared spectroscopy and imaging instrumentation and methodology. Neuroimage [Internet]. 2014;85:6–27. Available from: http://dx.doi.org/10.1016/j.neuroimage.2013.05.004

9.	Zhang X, Toronov VY, Fabiani M, Gratton G, Webb AG. The study of cerebral hemodynamic and neuronal response to visual stimulation using simultaneous NIR optical tomography and BOLD fMRI in humans. In: Bartels KE, Bass LS, de Riese WTW, Gregory KW, Hirschberg H, Katzir A, et al., editors. 2005. p. 566.

10.	Devor A, Boas DA, Einevoll GT, Buxton RB, Dale AM. Neuronal Basis of Non-Invasive Functional Imaging: From Microscopic Neurovascular Dynamics to BOLD fMRI. In 2012. p. 433–500.

11.	Aasted CM, Yücel MA, Cooper RJ, Dubb J, Tsuzuki D, Becerra L, et al. Anatomical guidance for functional near-infrared spectroscopy: AtlasViewer tutorial. Neurophotonics. 2015;2(2):020801.
